# Distinct Transcriptional and Anti-Mycobacterial Profiles of Peripheral Blood Monocytes Dependent on the Ratio of Monocytes: Lymphocytes

**DOI:** 10.1016/j.ebiom.2015.09.027

**Published:** 2015-09-24

**Authors:** Vivek Naranbhai, Helen A. Fletcher, Rachel Tanner, Matthew K. O'Shea, Helen McShane, Benjamin P. Fairfax, Julian C. Knight, Adrian V.S. Hill

**Affiliations:** a Wellcome Trust Centre for Human Genetics, Nuffield Department of Medicine, University of Oxford, Oxford, United Kingdom; b The Jenner Institute, Nuffield Department of Medicine, University of Oxford, Oxford, United Kingdom; c London School of Hygiene and Tropical Medicine, London, United Kingdom

**Keywords:** ML ratio, Monocyte, Transcriptome, Interferon signalling, Tuberculosis

## Abstract

The ratio of monocytes and lymphocytes (ML ratio) in peripheral blood is associated with tuberculosis and malaria disease risk and cancer and cardiovascular disease outcomes. We studied anti-mycobacterial function and the transcriptome of monocytes in relation to the ML ratio.

Mycobacterial growth inhibition assays of whole or sorted blood were performed and mycobacteria were enumerated by liquid culture. Transcriptomes of unstimulated CD14 + monocytes isolated by magnetic bead sorting were characterised by microarray. Transcript expression was tested for association with ML ratio calculated from leucocyte differential counts by linear regression.

The ML ratio was associated with mycobacterial growth in vitro (β = 2.23, SE 0.91, p = 0.02). Using sorted monocytes and lymphocytes, *in vivo* ML ratio (% variance explained R^2^ = 11%, p = 0.02) dominated over *in vitro* ratios (R^2^ = 5%, p = 0.10) in explaining mycobacterial growth. Expression of 906 genes was associated with the ML ratio and 53 with monocyte count alone. ML-ratio associated genes were enriched for type-I and -II interferon signalling (p = 1.2 × 10^− 8^), and for genes under transcriptional control of *IRF1*, *IRF2*, *RUNX1*, *RELA* and *ESRRB*. The ML-ratio-associated gene set was enriched in TB disease (3.11-fold, 95% CI: 2.28–4.19, p = 5.7 × 10^− 12^) and other inflammatory diseases including atopy, HIV, IBD and SLE.

The ML ratio is associated with distinct transcriptional and anti-mycobacterial profiles of monocytes that may explain the disease associations of the ML ratio.

## Highlights

• A pathophysiological basis for involvement of the ML ratio in infectious, malignant and cardiovascular diseases is unclear.
• Monocytes from individuals with high ML ratio have impaired control of mycobacterial growth and a distinctive transcriptome.
• These monocytes are characterised by enhanced interferon signalling, and intermediate transcription factors.
• The ML-ratio associated transcript signature is enriched in TB and other diseases including atopy, IBD and SLE.
• Ontogeny-specific function of monocytes may be the possible mechanism for the prognostic value of the ML ratio.

Enumeration of the absolute count of white blood cell subsets is one of the most frequently performed tests in clinical practice. Recently, elevation in the monocytes:lymphocytes (ML) ratio was linked to increased risk of tuberculosis and malaria disease, and poorer outcomes for many cancers and cardiovascular diseases. We sought to understand the mechanism of these disease associations. We found that the ML ratio reflects the functional capacity of monocytes and that genes that are differentially expressed in monocytes from donors with a high ML ratio are similar to those in other diseases such as HIV and inflammatory bowel disease. Because a specific type of blood-making stem cell regulates the ML ratio, it is plausible that the ratio reflects monocyte function because it is a clue to different monocyte origins. Our findings offer a mechanism for the predictive value of the ML ratio that may help improve its clinical utility.

## 1 Introduction

The diagnostic utility and prognostic value of leucocyte differential counts were appreciated contemporaneously with the development of microscopy methods to distinguish leucocyte subsets in 1880 (Ehrlich, 1880). A growing body of evidence suggests that the ratio of leucocyte subsets may be of similar, or greater, prognostic import than absolute counts. Although a full blood count is amongst the most frequently performed assays in clinical practice, the monocyte:lymphocyte (ML ratio) is not a widely-used parameter or biomarker in clinical care.

Florence Sabin and colleagues reported in the 1920s that the ML ratio was associated with progress and outcomes of mycobacterial infections in rabbits (Cunningham et al., 1925, Sabin et al., 1926, Doan and Sabin, 1930). Rediscovering this experimental work, we recently performed a series of prospective cohort studies of adults, infants and pregnant women in sub-Saharan Africa (Naranbhai et al., 2014a, Naranbhai et al., 2014b, Naranbhai et al., 2014a). In each study we observed that elevated ML ratio was associated with the risk of subsequent tuberculosis disease. We, and others, made similar observations for malaria (Warimwe et al., 2013a) and extended these to demonstrate that the ML ratio stratifies efficacy of the candidate anti-malaria RTS,S vaccine (Warimwe et al., 2013b). Other investigators have shown that an elevated ratio is associated with poor outcomes of nasopharyngeal carcinoma (Lin et al., 2014a, Li et al., 2013), diffuse large B-cell (Li et al., 2012, Li et al., 2014a, Li et al., 2014b, Rambaldi et al., 2013 Dec, Koh et al., 2014, Markovic et al., 2014 Mar, Porrata et al., 2014a, Porrata et al., 2014b, Watanabe et al., 2014, Wei et al., 2014, Yan-Li et al., 2014) and Hodgkin's (Koh et al., 2012, Porrata et al., 2012, Porrata et al., 2013a, Porrata et al., 2013b, Romano et al., 2012) lymphomas, multiple myeloma (Shin et al., 2013), clear-cell renal carcinoma (Hutterer et al., 2014), non-small cell lung cancer (Lin et al., 2014b), soft tissue sarcoma (Szkandera et al., 2014) and colon cancer (Stotz et al., 2014).

The neutrophil:lymphocyte (NL) ratio, another measure of the myeloid:lymphoid cell proportion, has also been reported to be associated with cardiovascular and cancer outcomes (Guthrie et al., 2013, Templeton et al., 2014, Wang et al., 2014a). Since the ML and NL ratios are strongly correlated it is unclear whether one is a better predictor than the other. The pathophysiologic basis for association of myeloid:lymphoid cell ratios across diseases remains unclear.

Here we studied pathophysiologic mechanisms for the association between the ML ratio and mycobacterial disease susceptibility. We studied anti-mycobacterial and transcriptomic profiles of monocytes from healthy adult donors and found that qualitative differences in monocyte function partially explain the ML ratio association with mycobacterial growth *ex vivo*. Whole-transcriptome analysis of monocytes suggests that the ML ratio (but not the monocyte count) is a marker of monocyte function and that an elevated ratio is associated with an enrichment of interferon-associated transcripts in monocytes. We show that the transcript signature of elevated ML ratio overlaps with mycobacterial and several other disease transcriptomic signatures such as atopy and inflammatory bowel disease. Taken together our data suggest that the ML ratio may be associated with disease by acting as a marker of monocyte function.

## 2 Materials and Methods

### 2.1 Study Participants

We recruited 144 healthy adult Caucasian volunteers in Oxford, United Kingdom (Fairfax et al., 2014). A healthcare professional (nurse or physician) conducted a verbal review of clinical history to determine eligibility based on the absence of any major chronic illness, current medication administration or symptoms of infection. The median age of the 144 recruits was 32 years (IQR 24–41) and 76 (53%) were female. For mycobacterial growth inhibition assays, we included individuals who had received BCG, were negative by interferon-gamma release assay (IGRA) and had no history of tuberculosis whereas for transcriptomic experiments, donors had no history of tuberculosis but their BCG history and IGRA status was not verified.

### 2.2 Ethics Statement

This study was approved by the Oxfordshire Research Ethics Committee (COREC reference 06/Q1605/55 & 10/H0505/3) and each individual gave written informed consent to participation.

### 2.3 Full Blood Counts

Leucocyte differential counts were performed at the Oxford Radcliffe Hospitals Pathology Laboratory, an accredited clinical laboratory, using standard procedures on a Sysmex automated haematology analyser. The ML ratio was calculated as the quotient of the absolute monocyte and lymphocyte counts.

### 2.4 Isolation of Monocytes

Whole blood was collected into sodium-heparin containing blood collection tubes (Becton Dickinson) and processed with 4–6 h after collection. The methods used in monocyte isolation have been previously described (Fairfax et al., 2014) but briefly, we isolated peripheral blood mononuclear cells by density gradient centrifugation of blood-diluted with Hanks Buffered Saline solution (HBSS, Life Technologies, UK) layered on Lymphoprep (Axis-Shield, Norway), and sorted CD14 + monocytes using magnetic-activated cell sorting (MACS, Miltenyi Biotech). The CD14- fraction was used in recreating ML ratios after counting cells by microscopy.

### 2.5 Mycobacterial Growth Inhibition Assays

Whole blood or mixed leucocyte growth inhibition assays were performed using the BACTEC mycobacteria growth indicator tube (MGIT) system (Becton Dickinson) as previously described (Wallis et al., 2001), with the exception that whole blood was incubated with BCG (Pasteur) for 96 h instead of 72 h and mixed leucocytes (monocytes + non-monocytes = total 1 × 10^6^ live cells) incubated with BCG Pasteur for 72 h. Growth inhibition was determined by calculating time to positivity (TTP) in the sample and TTP in the control (cell free culture) and converting to colony-forming units using a standard curve.

### 2.6 Gene Expression

RNA from 5 × 10^5^ sorted monocytes was extracted using the Qiagen RNAeasy kit (Qiagen, UK). RNA was quantified and integrity assessed using a Bioanalyser RNA 6000 Nano kit (Agilent, UK). Gene expression was quantified using the Illumina HumanHT-12 v4 BeadChip gene expression array platform with 47,231 probes according to the manufacturer's instructions. Samples were randomly placed across expression chips and run in a single batch. Gene expression data were normalized using random-spline normalization, transformed by variance-stabilising transformation and sample outliers were iteratively removed and normalization repeated. We excluded 8 individuals as sample outliers.

Probe sequences mapping to more than one genomic location or regions with underlying polymorphisms frequent in > 1% of the population were excluded from analysis (n = 18,220 probes). Only probes that were expressed and detected (GenomeStudio probe detection p < 0.01) in monocytes were included in the analysis.

### 2.7 Transcription Factor Binding Site Analysis

We used oPOSSUM (Kwon et al., 2012) v3.0 to perform single-site analysis with the JASPAR core reference and the following search parameters: conservation cutoff = 0.4, matrix score threshold = 85% and upstream and downstream sequence lengths 10,000 bp. Of 906 genes submitted for analysis 821 matched genes in the database.

### 2.8 Statistical Methods

Analyses were conducted in *R*, using the packages: limma, lumi and ‘vsn’,. For data presentation the ‘gridExtra’, ‘ggbio’ and ‘ggplot2’ packages were used. Linear regression adjusting for age and sex was performed using the lm function in R. Enrichment analyses were performed using the fisher.test function in R comparing the observed vs. expected overlap in gene lists.

Ingenuity Pathway Analysis where used, was performed using a background set of all human genes on the Ilumina array. A listing of external files and their sources is given in Supplementary Table 14. FDR where shown denotes the false discovery rate calculated using the Benjamini Hochberg procedure using the p.adjust function.

### 2.9 Accession Numbers

Gene expression data is available through ArrayExpress (E-MTAB-2232). The accession numbers of studies accessed through the ExpressionAtlas are shown in Supplementary Table 4.

## 3 Results

### 3.1 Qualitative Differences in Monocytes Dependent on ML Ratio Dominate Over Quantitative Differences in Explaining the ML Ratio Association With Mycobacterial Growth

**Fig. 1:**
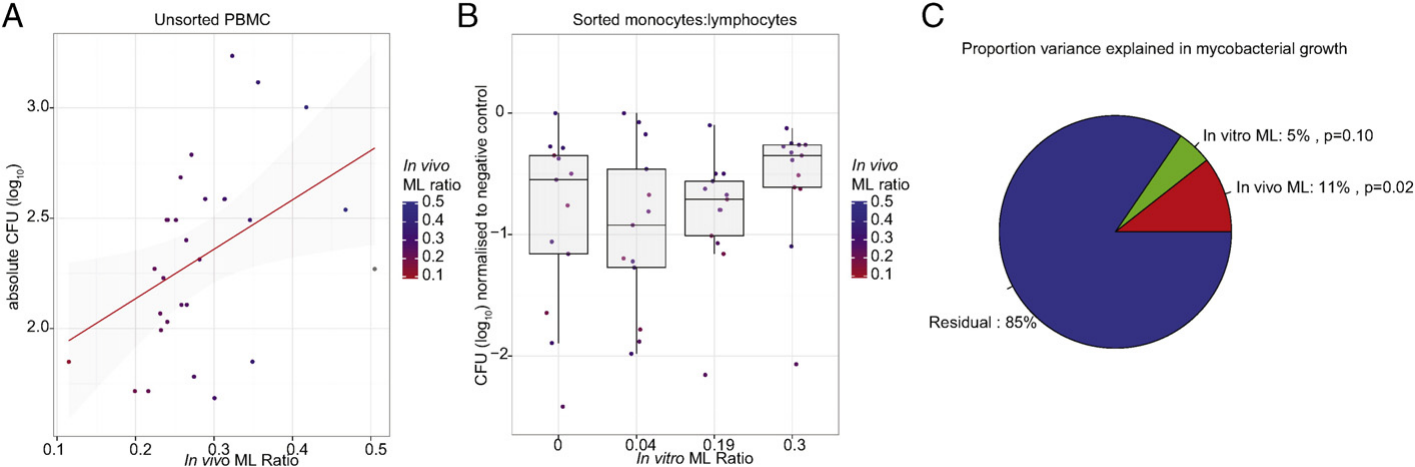
The ML ratio is associated with mycobacterial growth ex vivo, but the in vivo ML ratio of the donor dominates in explaining mycobacterial growth in a growth inhibition assay. (A) In a mycobacterial growth assay in which whole blood from healthy human adult donors (n = 29) was co-cultured with BCG Pasteur for 96 h and mycobacterial growth assessed by liquid culture, the ML ratio is associated with mycobacterial growth. Each donor is indicated by a dot that is coloured according to the in vivo ML ratio. A regression line and 95% CI is shown in red and grey respectively. (B) CD14 + monocytes and lymphocytes sorted from healthy human adult donors (n = 13) were combined in vitro at ratios that approximate the 25th (0.04), 50th (0.19) and 75th (0.30) centiles of the background population and cultured with BCG Pasteur for 72 h and mycobacterial growth assessed by liquid culture. Lymphocytes alone were added to the control tube (denoted as ML ratio = 0). Each donor is indicated by a dot that is coloured according to the in vivo ML ratio. Boxes denote the 25th, 50th and 75th centiles and whiskers extend to median ± 1.5 ∗ IQR. (C) In a multivariate linear regression model of data from part B, the in vivo ML ratio significantly explains 11% of the variance in mycobacterial growth whilst the in vitro ratio explains 5% of variance, albeit not statistically significant.

Recent epidemiological studies (Naranbhai et al., 2014b) suggest that elevated peripheral blood ML ratio is associated with risk of TB disease in adults, infants and pregnant women. To evaluate whether this finding is replicated *ex vivo*, we cultured whole blood from healthy adult donors with *BCG* (*Pasteur*) in a mycobacterial growth inhibition assay (Wallis et al., 2001). The *in vivo* ML ratio calculated from white cell differential counts in donors was significantly associated with enhanced mycobacterial growth *ex vivo* (β = 2.23, SE = 0.91, p = 0.02, Fig. 1A).

To distinguish between a quantitative effect of increasing numbers of monocytes providing more target cells to support mycobacterial growth, versus a qualitative effect of monocytes from individuals with higher ML ratio being relatively inferior at inhibiting mycobacterial growth, monocytes and lymphocytes were sorted from blood of 13 healthy adults, mixed at ratios *in vitro* that approximate the 25th, 50th and 75th centiles of ML ratios for adult populations and cultured with *BCG* (Pasteur). Higher *in vitro* ML ratio tended to be associated with elevated mycobacterial growth, however individuals with higher ML ratio *in vivo* supported greater mycobacterial growth regardless of the *in vitro* ML ratio that was created by mixing sorted cells (Fig. 1B) suggesting that qualitative differences play a role beyond quantitative differences. In a multivariate linear regression model, the *in vivo* ML ratio (β = 3.22, SE = 1.30, p = 0.02) but not the *in vitro* ML ratio (β = 1.12, SE = 0.67, p = 0.10) was significantly associated with mycobacterial growth (Fig. 1C) suggesting that qualitative effects dominate over quantitative effects in explaining monocyte capacity to control mycobacterial growth.

### 3.2 Transcriptional Profiles of Monocytes Dependent on the ML Ratio

To understand the qualitative differences in monocytes from individuals with varying ML ratio, we analysed data for CD14 + monocytes from 136 healthy European volunteers from Oxfordshire UK (68 male, 68 female, median age = 31.5 years, IQR 24–41) a subset of a cohort for whom we have generated transcriptome-wide profiling using the Illumina HT12v4 gene expression array (Fairfax et al., 2014). Donors were representative of a young healthy adult population in a region with low endemicity of mycobacterial disease. The median monocyte count was 0.4 (IQR 0.3–0.48) × 10^9^/L and median lymphocyte count 1.81 (IQR 1.54–2.12) × 10^9^/L. After quality control procedures (Methods), we evaluated 10,104 genes (13,393 probes) that were robustly detected by high fidelity probes and were expressed in > 5% donors.

We tested for association between the level of each transcript and either the monocyte count alone, lymphocyte count alone or the ML ratio (in each case adjusting for age and sex). The expression level of 53 genes (55 probes) was significantly associated (false discovery rate (FDR) < 0.05) with monocyte counts; 0 genes with lymphocyte count and 906 genes (991 probes) with ML ratio (Supplementary Table 1). For 51 of 53 genes (96%) whose expression was associated with monocyte counts, expression was also associated with the ML ratio; in these cases monocyte counts and ML ratio explained a similar proportion of overall gene expression variance (median 12.3% vs 13.4%, p = 0.6). Taken together these data suggest that the ML ratio reflects a larger degree of the monocyte transcriptome than monocyte counts alone, and confirms, as per the null expectation, that the monocyte transcriptome is independent of lymphocyte count.

**Fig. 2:**
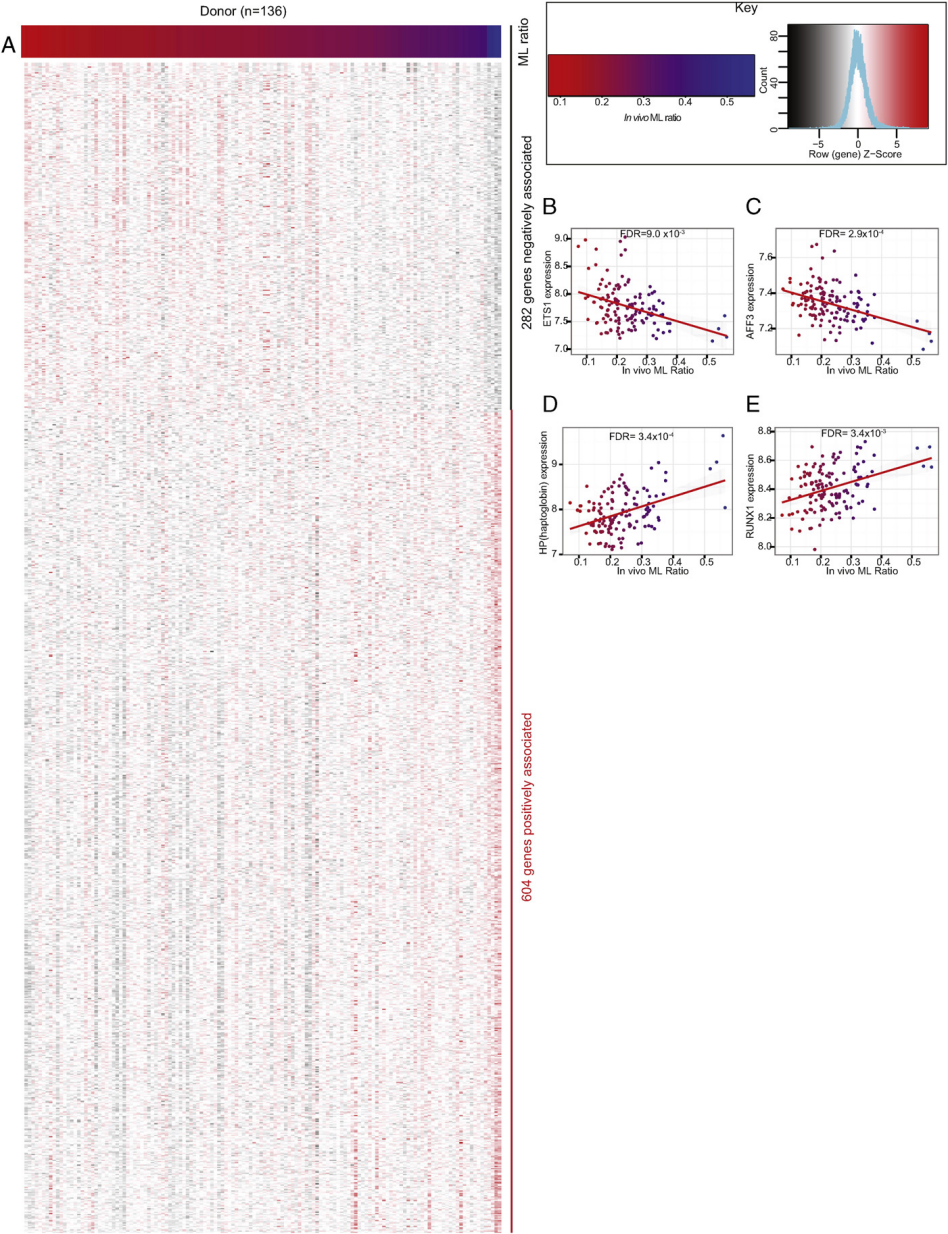
Monocyte transcriptomes are associated with the ML ratio of the human donor. (A) The ML ratio 139 individuals was associated with expression of 906 genes in CD14 + monocytes as shown in this heat map where donors are arranged according to ML ratio (along top), and direction of ML ratio-gene expression association (along right side). The key (top right) denotes colouring of individuals according to the ML ratio (top) and genes according to expression level (B-C) ETS1, encoding for a transcription factor involved in cytokine transcription, and AFF3, encoding for a transcription factor involved in lymphoid development, are examples of genes whose expression is negatively associated with ML ratio. In contrast (D-E) HP, which encodes for haptoglobin and RUNX1, encoding a transcription factor, are positively associated with ML ratio. In panels b–e, the regression line and 95% CI are shown in red and grey respectively.

Of 906 genes (991 probes) associated with the ML ratio (Fig. 2A), an elevated ML ratio was associated with reduced expression of 282 genes (296 probes) such as *ETS1* and *AFF3* (Fig. 2B–C), and elevated expression of 624 genes (695 probes) such as *HP* (haptoglobin) and *RUNX1* (Fig. 2D–E).

### 3.3 ML Ratio Associated Gene Transcripts are Enriched for Interferon Signalling

**Fig. 3:**
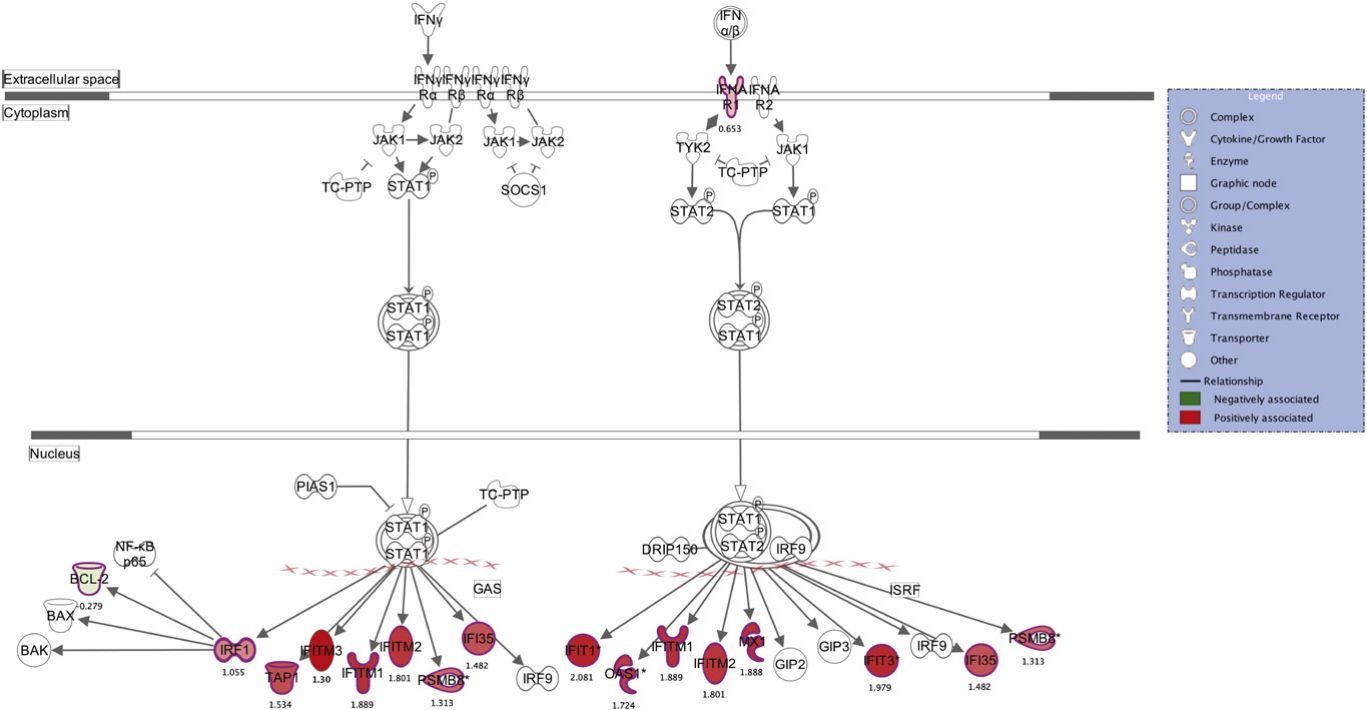
Monocyte genes associated with the ML ratio are enriched for genes in the interferon signalling pathway. The intensity of association with ML ratio is denoted in shades of red (increased) or green (decreased) proportionate to the level of expression and symbols denote the type of molecule. The text beneath each symbol denotes the effect size (beta) estimate between ML ratio and gene expression.

Interferon signalling (Fig. 3) was the most significantly enriched (p = 1.2 × 10^− 8^) canonical pathway (p < 0.05) (Supplementary Table 2). The five most significant upstream regulators were *IFNL1* (p = 5.1 × 10–23), *IFNA2* (p = 2.4 × 10–17), *TGM2* (p = 9.2 × 10–16), *MAPK1* (p = 5.1 × 10–15) and *IFNG* (p = 1.1 × 10–13). Genes downstream of *IFNL1*, *IFNA2*, *TGM2* and *IFNG* were enriched and markedly activated whilst genes downstream of *MAPK1* were enriched and markedly inhibited. Several other regulators involved in interferon signalling are similarly enriched (Supplementary Table 3) including TLR3, IFNA, TNF, IFN1B, IFNAR2 and STAT1.

### 3.4 Transcription Factor Binding Analysis

To further investigate the direction of association between ML ratio and monocyte transcriptomes, we considered whether the set of differentially expressed genes could be more parsimoniously explained by changes in expression of transcription factors. Of the 906 ML-ratio associated genes, 79 are human transcription factors, similar to the expected proportion (OR 0.85 95% CI 0.67–1.07) (Ravasi et al., 2010). Amongst the 38 transcription factors that were positively associated with ML ratio are *E2F4*, *STAT6*, *SP100*, *IRF1* and *IRF7*. Amongst the 41 transcription factors that were negatively associated with ML ratio are *ETS1*, *NOTCH2*, *KLF12* and *JUND* (Supplementary Table 1).

**Fig. 4:**
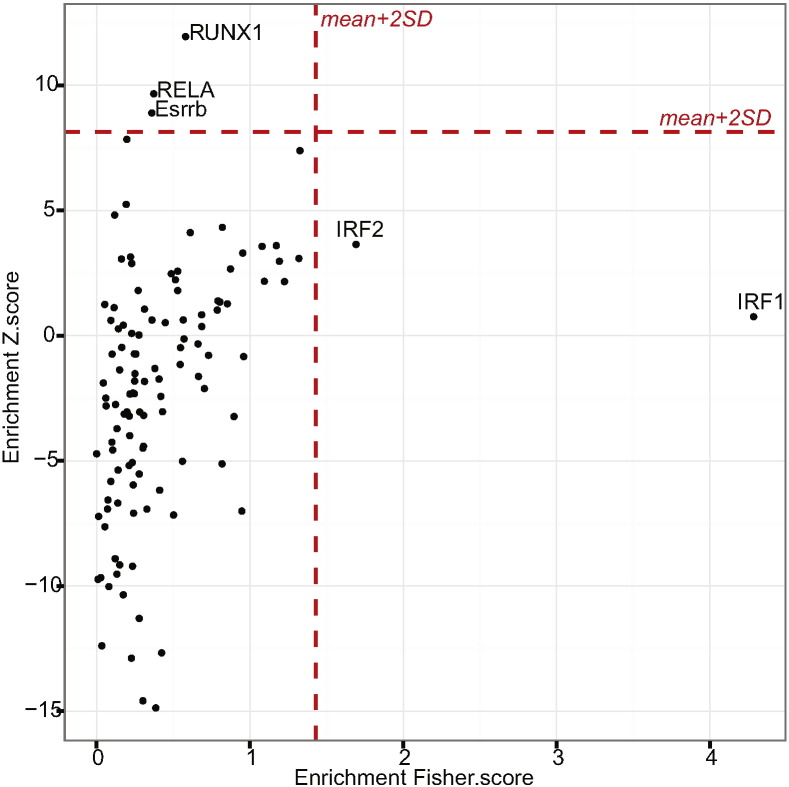
Monocyte genes associated with the ML ratio are enriched for transcription factor binding sites for IRF1, IRF2, RUNX1, RELA and Esrrb. The Fisher's exact test (x-axis) from oPossum disregards the number of times a TFBS occurs within a promoter in comparing observed vs expected TFBS frequency, whilst the z-score (y-axis) is a measure of the frequency of occurrence allowing for more than one TFBS motif within a gene promoted compared with the background expectation. The mean + 2SD is plotted in red. Genes with enrichment scores greater than the mean + 2SD are labelled.

To characterise this further, we proceeded to test whether ML ratio-associated genes were enriched for specific transcription factor binding consensus binding sites (TFBS) using oPOSSUM (Kwon et al., 2012). TFBS for *IRF1*/*IRF2*, *RUNX1*, *RELA* and *ESRRB* were enriched for in the genes associated with the ML ratio (Fig. 4). These data suggest that consequent upon upstream events such as interferon stimulation, transcription factors such as IRF1 may be involved in observed variation in monocyte transcriptomes.

### 3.5 Representation of ML Associated Transcripts in Human Diseases

In view of the apparent broad association of the ML ratio with malignant and non-malignant disease outcomes, we explored whether the set of ML associated genes in monocytes was enriched for amongst genes differentially expressed in blood in any of 151 human disease conditions curated in the Expression Atlas (Petryszak et al., 2014). For 19 disorders, there is significant enrichment for the ML transcript signature (Supplementary Table 4). These include atopy (FDR = 3.33 × 10^− 18^), HIV (FDR 1.44 × 10^− 10^), the inflammatory bowel diseases (FDR = 6.99 × 10^− 8^) ulcerative colitis and Crohn's disease, meningeal (FDR = 4.97 × 10^− 4^), pulmonary (FDR = 1.66 × 10^− 3^) and latent (FDR = 1.08 × 10^− 3^) tuberculosis, systemic lupus erythematosus (FDR = 4.36 × 10^− 3^) and type 1 diabetes mellitus (FDR = 5.1 × 10^− 3^).

### 3.6 ML Associated Transcripts are Enriched for Genes that are Differentially Expressed During TB Disease and for TB-Disease Susceptibility Associated Genes

On the basis of the epidemiologic associations, the in vitro data presented here and apparent enrichment for ML associated transcripts in a TB dataset curated in the Expression Atlas, we tested whether the ML associated gene set was similarly enriched in eight studies of tuberculosis not contained within the Expression Atlas that were recently collated and meta-analysed (Joosten et al., 2013). We observed a 3.50-fold (95% CI 2.64–4.61, p = 1.95 × 10^− 16^) enrichment for TB disease-associated genes amongst ML associated genes, confirming that the transcriptomic changes during TB disease partially consist of ML-associated transcripts. These data are consistent with the observed elevation of the ML ratio during active TB disease (J. Wang et al., 2014).

In the absence of transcriptomic signatures that associate with TB susceptibility we examined genes that have been implicated in TB susceptibility by candidate gene studies (Supplementary Table 5). Of 95 genes for which variants have been reportedly associated with TB, 56 are expressed and detected in monocytes and were tested for association with ML ratio. Eleven of 906 (1.2%) ML associated genes have been implicated in TB through candidate gene studies, a 2.5-fold (95% CI 1.16–4.93, p = 0.02) enrichment over background (45/9198, 0.049%). These were *CCL5*, *DEFB1*, *FCGR2A*, *HP*, *IRF1*, *MCL1*, *TAPBP*, *TIRAP*, *TLR4*, *TLR8* and *SP110*. This suggests that the ML ratio association with TB may, at least partly, overlap with genes in which genetic variation affects susceptibility.

## 4 Discussion

The relative frequency of myeloid and lymphoid cells is increasingly recognised as a variable distinct from its constituents, but the pathophysiologic basis of association remains unclear. Our observation of monocyte functional and transcriptional differences dependent on the ML ratio (but on neither constituent alone) suggests that qualitative differences in monocytes are better reflected by the ML ratio than by monocyte counts alone, potentially explaining epidemiologic associations of the ratio. The significant enrichment of interferon signalling we found supports a common role for type I and II interferons in altering the ML ratio and monocyte function sufficiently to explain altered disease course, consistent with the central role of interferons in mycobacterial and inflammatory diseases (Mayer-Barber et al., 2014). Additionally, alteration in monocyte function may alter crosstalk with lymphocytes and adaptive immune responses (Zhong and Yazdanbakhsh, 2013), hence changes in monocyte function alone may have repercussions on other aspects of the immune response that are detrimental. Finally, we demonstrate that the transcripts that are associated with the ML ratio are enriched in tuberculosis using external datasets and report evidence for a pathophysiologic role of ML-ratio associated genes in several other disease conditions.

Frequencies of leucocyte subsets in peripheral blood are a result of lineage commitment events during haematopoiesis, sequestration or egress into tissue and survival. Changes in cell fate are well described for lymphocytes, for example both bacterial and viral infection (Hotchkiss et al., 2013, Merekoulias et al., 2010, McClain et al., 2013) depress peripheral and splenic lymphocyte counts, and cause widespread lymphocyte apoptosis (Hotchkiss et al., 2013). Compared with lymphocytes, much less is known about reactive changes in monocyte number or function. Clonal analysis in mice demonstrates that haematopoietic stem cells have distinct lineage biases and can be classified as myeloid-biased, balanced or lymphoid-biased depending on the relative proportions of myeloid and lymphoid progeny they give rise to (Muller-Sieburg et al., 2012). In mice, myeloid-biased HSC differentiation has been shown to be under the control of IFN-γ (Matatall et al., 2014). In humans, myeloid-biased HSC accumulate with age and explain the relative increase in myeloid cells in blood with age (Pang et al., 2011). Therefore changes in ML ratio in blood are likely a marker of changes in the frequency of lineage-biased HSC. Intriguingly, mycobacterial infection has been shown to alter haematopoiesis through elevated IFN-γ levels (Baldridge et al., 2010). In murine models, ontogeny affects the functional capacity of the progeny of myeloid-biased HSC (Gekas and Graf, 2013, Muller-Sieburg et al., 2004). This finding is recapitulated in humans in whom granulocyte-monocyte progenitors give rise to distinct myeloid subsets, are increased in the circulation, and portend impaired outcomes in a range of solid organ malignancies (Wu et al., 2014). The causal pathway is difficult to unambiguously ascertain but positioning our data in this context suggest a model in which inflammatory signals - particularly interferons - affect the ML ratio through lineage-bias during haematopoiesis and this has a lasting effect on the transcriptional and functional profile of the monocyte progeny.

Our study is limited in ability to exhaustively examine all potential mechanisms in operation. We studied only CD14 + monocytes and it is possible that changes in other monocyte subsets may occur. Our evaluation of ML ratio transcripts in other diseases is limited to bioinformatic overlap with data curated in the Expression Atlas. Further studies will be required to confirm a role, if any, of the ML ratio in these disorders as it is plausible that the overlap is driven by common involvement of interferon pathways in these disorders. Finally, our study suggests that the ML ratio and monocyte function may be a consequence of inflammatory processes acting, in part, through interferons. Identifying upstream drivers and feedback loops of these inflammatory processes requires further study, but may yield understanding of how to clinically address the pathophysiological basis of the disease association.

Collectively our data suggest that elevation in the ML ratio is associated with transcriptional changes in monocytes that may account for impaired anti-mycobacterial profiles of monocytes, potentially explaining some pathophysiologic disease associations of the ML ratio.

## Author Contributions

VN, BPF, JCK and AVSH designed the study. VN conceived the idea for this study, conducted the analysis and wrote the first draft of the manuscript. VN, BPF, HAF, RT and MOS performed wet lab experiments for this study and/or provided expertise and reagents. AVSH, JCK, BPF and HMS supervised the analysis and conduct of this study. All the authors had access to the data and the final decision to submit was made collectively by the authors.

## Acknowledgements/funding

We thank the volunteers for their participation in this study, Dr. Matthew Neville and Sr Jane Cheeseman for the assistance in enrolling participants and Dr. Seiko Makino for the technical assistance in isolation of monocytes. We thank Dr. Robert Petryszak for generating the gene lists for diseases curated in the ExpressionAtlas. We thank the volunteers from the Oxford Biobank(www.oxfordbiobank.org.uk), a NIHR Oxford Biomedical Research Centre, for their participation. The Oxford Biobank is also part of the NIHR National Bioresource which supported the recalling process of the volunteers. This work was supported by the Wellcome Trust (grants 074318, 088891 and 090532/Z/09/Z [core facilities Wellcome Trust Centre for Human Genetics including High-Throughput Genomics Group]), the European Research Council under the European Union's Seventh Framework Programme (FP7/2007–2013)/ERC Grant agreement no. 281824 (J.C.K.) and the NIHR Oxford Biomedical Research Centre. VN is supported by the Rhodes Trust, HMcS is a Wellcome Trust Senior Clinical Research Fellow, BPF is a Wellcome Trust MB/PHD fellow and AVSH a Wellcome Trust Senior Investigator. The funders had no role in the study design, data collection, data analysis, interpretation, and writing of the report.

## Appendix A Supplementary data

Supplementary Table 1: Results of association analysis of gene expression in monocytes with the host ML ratio or monocyte count. Gene, predicting parameter, association statistics and annotation of subcellular location and whether a transcription factor is shown. Supplementary Table 2: Canonical pathways enriched amongst monocyte genes whose expression is associated with the ML-ratio from ingenuity pathway analysis. Supplementary Table 3: Upstream regulators of ML-ratio associated transcriptomic changes from ingenuity pathway analysis. Supplementary Table 4: ML-ratio associated signature enrichment amongst genes associated with other diseases. Supplementary Table 5: Genes with variants that are reported to be associated with TB disease susceptibility and the association between their transcript levels in monocytes and ML ratio.
